# A Hybrid Prediction Method for Plant lncRNA-Protein Interaction

**DOI:** 10.3390/cells8060521

**Published:** 2019-05-30

**Authors:** Jael Sanyanda Wekesa, Yushi Luan, Ming Chen, Jun Meng

**Affiliations:** 1 School of Computer Science and Technology, Dalian University of Technology, Dalian 116023, Liaoning, China; jael@mail.dlut.edu.cn; 2 Department of Information Technology, Jomo Kenyatta University of Agriculture and Technology, Nairobi 62000-00200, Kenya; 3 School of Bioengineering, Dalian University of Technology, Dalian 116023, Liaoning, China; luanyush@dlut.edu.cn; 4 College of Life Sciences, Zhejiang University, Hangzhou 310058, Zhejiang, China; mchen@zju.edu.cn

**Keywords:** autoencoder, random forest, light gradient boosting machine, hybrid, lncRNA-protein interaction, plant

## Abstract

Long non-protein-coding RNAs (lncRNAs) identification and analysis are pervasive in transcriptome studies due to their roles in biological processes. In particular, lncRNA-protein interaction has plausible relevance to gene expression regulation and in cellular processes such as pathogen resistance in plants. While lncRNA-protein interaction has been studied in animals, there has yet to be extensive research in plants. In this paper, we propose a novel plant lncRNA-protein interaction prediction method, namely PLRPIM, which combines deep learning and shallow machine learning methods. The selection of an optimal feature subset and subsequent efficient compression are significant challenges for deep learning models. The proposed method adopts *k*-mer and extracts high-level abstraction sequence-based features using stacked sparse autoencoder. Based on the extracted features, the fusion of random forest (RF) and light gradient boosting machine (LGBM) is used to build the prediction model. The performances are evaluated on *Arabidopsis thaliana* and *Zea mays* datasets. Results from experiments demonstrate PLRPIM’s superiority compared with other prediction tools on the two datasets. Based on 5-fold cross-validation, we obtain 89.98% and 93.44% accuracy, 0.954 and 0.982 AUC for *Arabidopsis thaliana* and *Zea mays,* respectively. PLRPIM predicts potential lncRNA-protein interaction pairs effectively, which can facilitate lncRNA related research including function prediction.

## 1. Introduction

Ribonucleic acid (RNA) is a polymeric single-stranded molecule that constitutes living cells alongside deoxyribonucleic acid (DNA) and protein. Long non-coding RNAs (lncRNAs) are broad and myriad group of endogenous single-stranded polynucleotides non-protein coding transcripts with greater than 200 nucleotides sequence length [1]. Previously, lncRNAs were thought to be transcriptional noise because they are less efficiently spliced compared with messenger RNAs (mRNAs). Nevertheless, accumulated experimental evidence has suggested that lncRNAs have indispensable roles in development, hormone-dependent signaling, and stress responses in plants [2,3]. For example, lncRNA COLDAIR in *Arabidopsis thaliana* regulates flowering time and floral organ formation during vernalization [4]. Functional roles of lncRNAs in plants are lagging behind compared with other organisms such as animals, bacteria, and viruses [5]. The mechanism underlying the functions of lncRNAs is a captivating area of research. More and more studies have indicated that regulation of transcriptional, post-transcription processes and new insights into potential functions can be elucidated by lncRNAs binding with proteins [6]. Additionally, information about these interactions can hint at molecular causes of diseases in plants and animals [7]. lncRNA-protein interactions have been revealed by various experimental methods. For instance, using a fluorescence resonance energy transfer (FRET) approach coupled with fluorescence lifetime imaging microscopy (FLIM) to detect RNA-protein interactions in plant leaves [8]. Other experimental methods such as RNA-protein pull-down assays and RNA immunoprecipitation protocol have been adapted for detecting RNAs and their protein interaction partners in plants [9,10]. However, these wet-lab experimental methods are time-consuming, labor-intensive, and relatively few lncRNA-protein associations have experimental proof. Therefore, computational prediction of RNA and protein interaction partners to complement experimental approaches has become increasingly crucial.

To hasten research, many genome databases have been set up. The databases and web-based platforms available for human and mammals blend lncRNA information including functional annotation, phylogenetic conservation, associations with diseases, and relations between lncRNAs with other RNAs and proteins [5]. However, databases for plant lncRNAs are incomprehensive [5]. Over the last decade, many plant lncRNAs have been discovered by both experimental and computational screening [11]. Computational tools such as LncFinder [12], PlncPRO [13], PlantRNA Sniffer [14], and LncADeep [15] have produced a huge influx of plant lncRNA data. Two main approaches prevalent across lncRNA-protein interaction methods are feature-based and similarity-based [16]. Similarity-based methods assume that lncRNAs with similar functions interact with similar proteins, a concept known as ‘guilt-by-association’. Feature-based methods extract features from input data and employ methods such as support vector machine (SVM) to predict interactions between lncRNAs and proteins. In previous research, lncRNA-protein association prediction is based on biological characteristics such as sequence, structure, localization, and genomic context. For example, catRAPID [17] used physicochemical properties including secondary structure, hydrogen bonds, and van der Waals forces between proteins and lncRNAs to assess the tendency of interaction. Lu et al. developed lncPro [18] for lncRNA-protein interaction based on Van der Waals propensities, hydrogen bonding, and secondary structures. RPI-Pred [19] predicted ncRNA-protein interactions by integrating 3D structural and sequence features. LPI-ETSLP [20], a semi-supervised method based on sequence data, revealed lncRNA-protein association using eigenvalue transformation without the need for negative samples. Zhao et al. developed IRWNRLPI [21], a method that integrates random walk and neighborhood regularized logistic matrix factorization for forecasting lncRNA-protein association. Moreover, many network-based methods have been proposed to predict lncRNA-protein interaction based on the integration of heterogeneous networks including LPIHN, RWR, and LPI-NRLMF [22,23,24].

Traditional methods use hand crafted features that consume more time, require strong domain knowledge, and are problem-dependent. Therefore, it is immensely beneficial to develop a feature extraction method that is multi-layered (deep) to enhance representation power and provide insight into complex features [25]. Deep learning (DL) methods compute high-level representations from raw input. These models have been broadly utilized by researchers to understand the molecular mechanism in human and plant diseases [26,27,28]. As the hottest sub-field of machine learning, DL methods extract intrinsic features through multilayer architectures such as autoencoders (AE), convolutional neural networks (CNN), and recurrent neural networks (RNN) [29]. In computer vision, speech recognition, and text processing, DL has been observed to perform better than other popular machine learning methods [30]. It has also been successfully implemented in bioinformatics [31,32,33]. For example, DeepBind [31] is a DL-based model developed to identify sequence specificities of DNA and RNA binding protein. Similarly, DeepSEA [32] predicted noncoding-variant effect from chromatin-profiling sequences using DL. Recently, Pan et al. [34] predicted RNA-protein interaction by local-global deep CNN. Despite their significantly outstanding performance, selection of an optimal feature subset and subsequent efficient compression are still significant challenges in the existing methods. Therefore, deriving the most salient features is essential for improving performance. This can be achieved by imposing sparsity constraints including non-negativity on weights, weight decay regularization, and adding *l*_1_/*l*_2_-regularization terms to loss function [35,36]. *l*_1_/*l*_2_-regularization terms, referred to as mixed norm, minimizes reconstruction error in the AE model during training. Other constraints associated with AE include randomly corrupted data inputs, referred to as denoising AEs [37].

The most appealing characteristic of DL is the ability to extract high-level features to leverage the large number of unlabeled instances. Based on the extracted features, classifiers such as support vector machine (SVM) and random forest (RF) are used to build the prediction model. However, while a single classification model performs well, fusing multiple models through ensemble learning improves performance. An ensemble learning method is a meta-algorithm that trains several baseline models and combines them into a single predictive model. Apart from improved classification, ensemble methods perform well in problems involving noisy and imbalanced datasets [38]. Classification strengths of individual base classifiers selected for construction of the overall ensemble model lead to more accurate predictive performance. In 2011, a method named RPISeq [39] extracted 3-mer and 4-mer sequence features to train RF and SVM models for prediction of protein-RNA interaction. Then, Wang et al. [40] presented a model that predicted interactions between proteins and RNAs based on Naive Bayes (NB) and an extended NB classifier. In 2016, Pan et al. developed IPMiner, a sequence-based method for predicting lncRNA-protein interactions based on stacked autoencoder [41]. Yi et al. proposed RPI-SAN for lncRNA-protein interaction based on stacked autoencoder and RF [42]. lncLocator [43] predicted lncRNA subcellular localizations based on an ensemble of SVM and RF classifiers. A recent tool termed HLPI-Ensemble (human lncRNA-protein interaction) was proposed to predict human lncRNA-protein interactions based on SVM, extreme gradient boost (XGB), and RF [44]. For cancer prediction, a model based on DL with an ensemble of three classifiers was recently proposed by Xiao et al. [45].

In this study, we present a hybrid learning-based method, termed PLRPIM. Our objective is to predict lncRNA-protein interactions for plant species and demonstrate its significance in lncRNA annotation since most plant lncRNAs functions are unknown. Intrinsic high-level features are extracted by stacked sparse AE, a deep generative model. We use *k*-mer sparse matrices to represent lncRNA and protein sequences. Finally, the extracted features are fed into an ensemble of two classifier models, RF and light gradient boosting machine (LGBM) for prediction. The final outcome is computed by implementing a majority voting mechanism to the individual classification outcomes. The main contributions are: 1) We apply DL to extract high-level abstraction features from lncRNA and protein sequences; 2) adopt dropout, ReLU activation function, and *l*_1_/*l*_2_-regularization sparsity penalties to significantly reduce overfitting and improve performance; 3) fully exploit sequence information from *k*-mer and heterogeneous ensemble strategy to further improve prediction accuracy. Experimental results on two datasets including *Arabidopsis thaliana* and *Zea mays* show that our method performs better than other state-of-the-art methods such as RPISeq-RF, RPI-SAN, and IPMiner.

## 2. Materials and Methods

### 2.1. Dataset

We used sequence data from the plant lncRNA database (PlncRNADB) of lncRNA and their RNA-binding protein partners. The lncRNA-protein interaction data of *Arabidopsis thaliana* and *Zea mays* are downloaded from the website: http://bis.zju.edu.cn/PlncRNADB. *Arabidopsis thaliana* has 390 lncRNAs and 163 RNA-binding proteins whereas *Zea mays* has 1107 lncRNAs and 190 RNA-binding proteins. The dataset for *Arabidopsis thaliana* contained 948 positive samples (interactive pairs) and the *Zea mays* dataset contained 22,133 positive samples. The same number of negative pairs (948 and 22,133) were generated through randomly pairing proteins with lncRNAs and further removing the existing positive pairs [39]. Then, we used 20% of the data as the hold-out test set. Details of the datasets are shown in Table 1.

### 2.2. Interaction between LncRNA and Protein

We consider sequence features that are likely to be significant in predicting protein-lncRNA interactions. The complex molecular features include the number of atoms, hydrogen bonds, evolutionary conservation score, mutual interaction propensity, and electrostatic charges [46]. The interaction information between lncRNA and protein is derived from biological knowledge. The positive set consists of a concatenation of lncRNA-protein interacting pair *l_i_* and *p_j_* while the negative set consists of non-interacting pairs. To obtain lncRNA-protein interactions, we denote an interaction matrix *Y* as *Y*(*l_i_, p_j_*). *Y*(*l_i_, p_j_*) ∊ {0,1} where 1 indicates an interaction between *l_i_* and *p_j_* and 0 indicates no interaction. To define interaction profiles of lncRNA and protein, we use variables *m = SW*(*l_i_,* *l_j_*) and *n = SW*(*p_i_,* *p_j_*), where *SW* refers to Smith-Waterman (SW) algorithm [21]. *SW*(*l_i_*, *l_j_*) and *SW*(*p_i_,* *p_j_*) are the measures of similarity between sequence of lncRNA *l_i_* and *l_j_* and sequences of protein *p_i_* and *p_j_*. The underlying presumption of *m* and *n* is that lncRNAs collaboratively interact with akin protein partners and vice versa.

### 2.3. Sequence Feature Encoding

Feature encoding is a key task during construction of a sequence-based machine learning model for prediction. Sequence feature encoding in this work is based on *n*-gram technique, also known as *k*-mer of length *k* where lncRNA is represented by (*k*_1_ = 1, 2, 3, 4) and proteins (*k*_2_ = 1, 2, 3) descriptors. In our experiments, protein and lncRNA sequences are coded using two methods: 1) Autocovariance and 2) conjoint triad. We investigate sequence similarities between lncRNAs and proteins to determine the combination of features. A feature vector of a varied length of size *m* for lncRNAs and *n* for proteins is generated. We calculate sequence similarity in lncRNAs using sequence information. Let *S*_lncRNA_ represent a set of lncRNA sequence of interest. In order to compute similarities between lncRNA *l_i_* and *l_j_*, the following formula is used:

(1) $$S_{\ln cRNA}(l_{i},l_{j})=\frac{SW(l_{i},l_{j})}{\max(SW(l_{i},l_{i}),SW(l_{j},l_{j}))}$$

We encode amino acid for protein sequences by natural numbers selected randomly. A feature vector of a varied length of size *n* is generated. Protein sequences are computationally analyzed by mapping amino acid bases into the numeric values. Let *S*_protein_ represent a set of the protein sequence of interest. Given proteins *i* and *j*, we find the similarity between them based on SW dynamic programming algorithm using the following formula:

(2) $$S_{\mathrm{Pr}otein}(p_{i},p_{j})=\frac{SW(p_{i},p_{j})}{\max(SW(p_{i},p_{i}),SW(p_{j},p_{j}))}$$

### 2.4. The PLRPIM Pipeline

**Figure 1:**
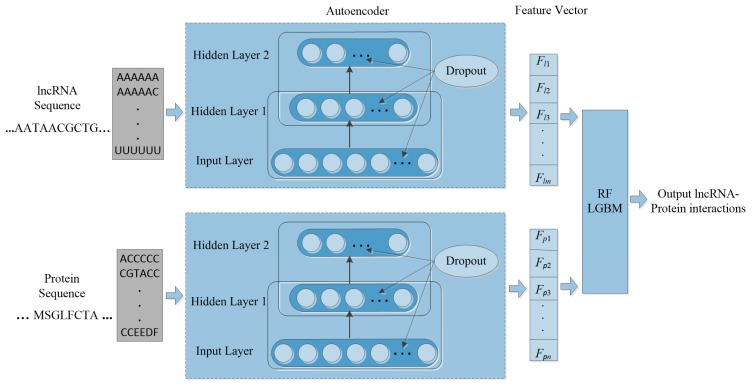
The workflow of PLRPIM model.

Machine learning is applied in RNA-protein interaction (RPI) prediction to address the challenge of sparsity of known RPIs. The essence is to select effective feature combinations. In this study, we present a stacked AE framework with sparse constraints for lncRNA-protein interaction prediction, namely PLRPIM. PLRPIM is a neural network model described as follows. First, each known lncRNA-protein interaction pair is assigned 1 and 0 to non-interacting pairs. Secondly, AE maps a pair of input protein and lncRNA sequences to representations. Then, the integrated lncRNA and protein feature matrices ${\left\{X_{i}\right\}}_{i=1}^{m}$ and ${\left\{W_{i}\right\}}_{i=1}^{n}$ are transformed into feature vectors. Feature vector for lncRNA is represented as *F_lncRNA_* = {*F_l_*_1_, *F_l_*_2_,…, *F_lm_*} and *F_protein_* = {*F_p_*_1_, *F_p_*_2_,…, *F_pn_*} for protein. Therefore, two subnetworks of lncRNA and protein are generated. Finally, a softmax layer is used to merge the two separate feature vectors and integrate them into a fully connected neural network to predict the label of each pair. lncRNA-protein interaction matrix *M_ij_* generated from predicted lncRNA-protein interaction matrix is the expected output matrix. To improve the performance of the proposed method in terms of robustness and accuracy, we adopt an integration strategy. We merge results from two machine-learning methods, RF and LGBM, to construct the prediction framework. The technical workflow of the proposed method is given in Figure 1.

#### 2.4.1. Feature Extraction

Features are the sequence-based consolidated attributes of lncRNAs and proteins encoded into numeric vectors that are used in the prediction. In this work, we selected the *k*-mer model to extract features from lncRNA and protein, where the length of genetic sequence subset *S* is represented by an integer *k.* The *k*-mer method has been successfully used in extracting features from RNAs and amino acid composition of proteins based on local sequence patterns. RNA sequence consists of symbols from four alphabets, A, G, C, and T. RNA sequences are represented as the occurrence frequencies of *k*-mers following the principle of complementary pairing [47]. Each *k*-mer pattern is represented as 4*^k^* for each *k* value, where 4 is the number of RNA sequence nucleotides. For example, 16 patterns (e.g., “AA”,”AC”,”AT”,…) can be obtained when *k* = 2. We extracted 4^4^ number of lncRNA sequence features by utilizing the *k*-mer scheme. Each lncRNA sequence is converted into a *k*-mer sparse matrix from which feature vectors are extracted. A protein chain has amino acids with 20 alphabets (A, C, D, E, F, G, H, I, K, L, M, N, P, Q, R, S, T, V, W, Y).

To obtain highly efficient features, we extracted a set of 599 descriptors from feature vectors encoded by various properties of lncRNAs and proteins. A total of 256 features are obtained from lncRNA sequence and 343 amino acid descriptors from protein sequence. We extract 4-mer sparse matrix of RNA sequence (A, C, G, T) by searching each sequence from left to right and obtain 256 (4 × 4 × 4 × 4) feature map. For the protein sequences, we partition amino acid compositions based on their chemical similarity. Seven groups of physicochemical properties {Val, Gly, Ala}, {Phe, Pro, Leu, Ile}, {Ser, Tyr, Met, Thr}, {His, Asn, Tpr, Gln}, {Arg, Lys}, {Glu, Asp}, and {Cys} of protein sequences were assigned numbers based on dipole moments (<1.0, <1.0, (1.0, 2.0), (2.0, 3.0), >3.0, >3.0, and <1.0) and their chain volumes (<50, >50, >50, >50, >50, >50, and <50) [19,41]. We extracted 3-mer counts of group labels to create a sparse matrix of 343 (7 × 7 × 7) feature map.

#### 2.4.2. Hybrid Learning Method

**Figure 2:**
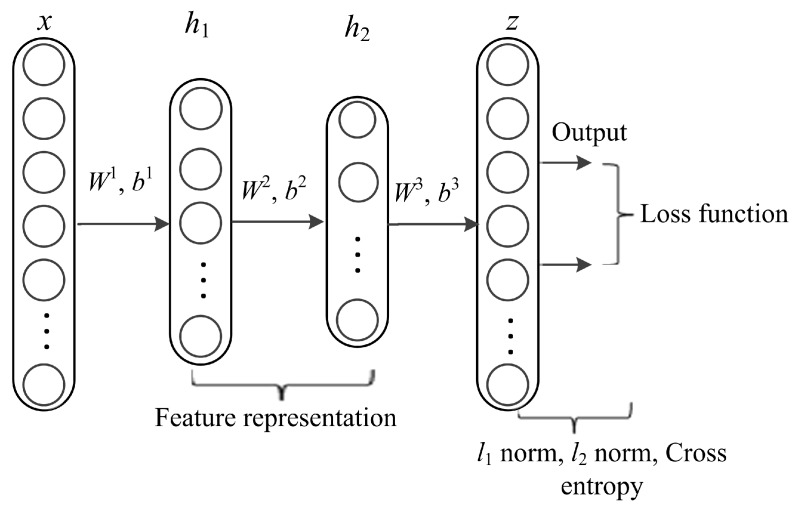
The network structure of constrained stacked autoencoders (AE).

DL architectures capture complex correlations in feature representations for diverse prediction tasks. The utmost significant property is learning features at multiple abstraction levels and feature concatenation within and across datasets. The core idea of stacked AE via sparse representations is to effectively obtain descriptors of data as linear projections that maximizes the correlation between features. AE is a kind of artificial neural network designed to extract and generate abstract features of high-dimensional data. It applies unsupervised learning algorithm to construct hidden structures from unlabeled data. The process of AE training consists of two components, an encoder and decoder. The encoder (function *f*) is used for mapping the input data (*x, y*) into latent representation and the decoder (function *g*) maps the encoded features to reconstruct input data from the latent representation. In this work, we formulate lncRNA-protein interaction prediction as a binary classification problem. We used constrained stacked AE (CSAE) network for DL and classification of training dataset as shown in Figure 2. The sparsity constraints extract the most informative features for optimal sample selection. Stacked AEs have greater expressive power and the successive layers of representations capture a hierarchical grouping of the input similar to convolution and pooling operations in CNN. Notations *W =* {*W^1^*, *W^2^*,*…*, *W^nl^*} and *b* = {*b*^1^*, b*^2^*, …, b^nl^*} denote the AE parameters. Weight matrix *W* denoted as $W_{ij}^{l}$ is the weight associated with the connection between neuron unit *j* in layer *l*-1 and neuron unit *i* in layer *l*, where *j* = 1,2,…, *sl* - 1, *i* = 1,2,…, *sl* and *l* = 2,3,…,*nl*. *sl* - 1 represents the output of the previous layer while *nl* represents the number of layers in the network. Bias vector is denoted as $b_{i}^{l}$ where *i* = 1, 2,…,*sl* and *l =* 2, 3,…,*nl* denotes the bias of neuron unit *i* in layer *l*. The training set is defined as {(*x*^1^*, y*^1^), (*x*^2^*,y*^2^),…,(*x^n^*, *y^n^*)} of *n* data samples. Suppose *x* is the input data of dimension *d*, the AE maps *x* to *y* as shown in the following formula:

(3) y=f(Wx+ b)

where *f* is the encoding function and *Wx* is a weight matrix that maps the output of *x* to a hidden-space. After mapping *x* to *y*, the output of the encoder (*y*) is mapped back to form *z,* which is the transformation of *x* with the same shape as *x*. The reconstruction is formulated as follow:

(4) $$z=g(W^{T}y+b^{\prime })$$

where *g* is the decoding function, non-linear function *W^T^* is the weighting matrix for transformation, and *b′* is the transformation bias. The transformation error is estimated using squared error between *x* and *z.* To minimize and optimize the reconstruction error, parameters *w* and *b* are adjusted using stochastic gradient descent (SGD) [48]. Cross-entropy, the most common type of loss function, is used to minimize reconstruction error by AE. We use 256, 128, and 64 layers that are fully connected. We model the neurons output *f*(*x*) using rectified linear unit (ReLU) activation function:

(5) f(x)=max(x,0).

We trained the AEs by adding dropout layers. Dropout layers regularize the model to avoid the risk of overfitting by randomly leaving out some neuron units.

#### 2.4.3. Training of PLRPIM Model with Mixed Norm Constraint

Inducing sparse constraints in AEs including weight decay and norm penalties has been successfully implemented to achieve high discriminative power of models. Inspired by [35,36], we implement mixed norm regularizer constraints to train stacked AE. The sparse *l*_1_ and *l*_2_ penalties for the weights are defined as follows:

(6) $$l_{1}=\frac{\lambda }{2}\sum_{i=1}^{m}\left\|W_{i,j}^{l}\right\|,$$

(7) $$l_{2}=\frac{\lambda }{2}{\sum_{i=1}^{m}\left\|W_{i,j}^{l}\right\|}^{2}$$

where $W_{i,j}^{l}$ denotes the connection between neuron unit *i* and *j* in layer *l*.

The proposed model is multi-layered and is trained in three phases. We use feed forward to optimize weights of the networks and adopt backpropagation in the last phase of training the network. The stacked AE-based generated networks are pre-trained before using backpropagation algorithm. The layers in the model include an input layer *x* with data points {(*x*^1^*,y*^1^*), (x*^2^*,y*^2^*),…,(x^n^,y^n^) }*, hidden layers {*h*_1_*, h*_2_}, and an output layer *z* (Figure 2). The features obtained from the hidden layers are used to reconstruct the input data and train the network. The first phase of the training process (pre-training) is realized by minimizing an objective function using cross-entropy (*C*) to avoid overfitting using Formulas (8) and (9) as follows:

(8) C=−(ylogz+(1−y)log(1−z))

(9) $$\underset{w,b}{\min}\left[\sum_{i=1}^{m}{\left(z\;-\;x\right)}^{2}+\beta \sum_{j=1}^{k}C\right]$$

where *y* are the labels of training examples, *z* represents the predicted output from input data *x*, *m* is the index of the number of training examples, and *k* is the index of the number of features. *β* is the trade-off parameter used to control overfitting. The second phase is made up of two hidden layers (*h*_1_ and *h*_2_). The third phase is the output layer, which receives input from the second hidden layer. In this phase, first, the weight and bias parameters (*W*^3^ and *b*^3^) are initialized. Then, a backpropagation algorithm is adapted to compute derivatives of the objective function and tune weights.

We apply mixed norm regularizer (*l*_1_/*l*_2_) with sparsity coefficient *λ* to minimize reconstruction error. The mixed norm regularizer is based on the assumption that descriptors from a set of training examples exhibit similar sparsity patterns in the feature map [49]. Formula (10) shows the computation of the mixed norm regularizer:

(10) $$\underset{w,b}{\min}\left[\sum_{i=1}^{m}{\left\|x-z\right\|}^{2}+\beta \sum_{j=1}^{k}C+\frac{\lambda }{2}{\left\|W\right\|}_{1,2}\right].$$

The parameter *β* is used for regularization, *λ* ≥ 0 is the weights sparsity term, ${\left\|W\right\|}_{1,2}$ represent *l*_1_ and *l*_2_ norms defined in Formulas (6) and (7) where ${\left\|W\right\|}_{1}=\sum_{i=1}^{m}\left\|W_{ij}^{l}\right\|$ and ${\left\|W\right\|}_{2}={\sum_{i=1}^{m}\left\|W_{ij}^{l}\right\|}^{2}$. Formula (10) minimizes the average reconstruction error, increase sparsity of latent layer activation, and reduce the number of non-negative weights.

#### 2.4.4. Shallow Classification Models

A vast number of classical machine learning methods have been employed in research and real-world systems to solve classification and regression problems. For RNA-protein interaction problem, SVM and RF shallow classification methods have previously been used [41]. Therefore, we consider these two and four other methods, adaptive boosting (Adaboost), XGB, LGBM, and decision tree (DT) to compare the performance of the single with merged classifiers. We assessed the prediction performance of the six commonly used classification methods. For most artificial intelligence applications, all the classification models are of high accuracy. However, each method may outperform others in different cases. We exemplify determining the most relevant kernel (radial basis function, linear, and polynomial) function challenge for SVM, while RF and gradient boosting decision tree (GBDT) may produce better classification results for the categories with more samples [45]. Therefore, a method that takes advantage of more than one classifier leads to superior prediction performance in practice.

The most widely used method for RNA-protein interaction prediction is SVM [50]. SVM is non-probabilistic classifier that maps input data points into a high-dimensional feature space. Other methods are ensemble methods, which are categorized into three classes: Bagging, boosting, and stacking. They combine several learning methods to obtain a predictive model with improved performance. They include RF, Adaboost, XGB, and LGBM. Given *n* models, *f_i_* is averaged into an ensemble *e*:

(11) $$e(x)=\frac{1}{n}\sum_{i=1}^{n}f_{i}(x).$$

RF is a widely used shallow machine learning method that combine decision tree predictors following the bagging technique [45]. In this model, the class that receives majority votes from trees in the forest is considered the output result. This protocol relies on creating *n* number of models and averaging predictions of all models for a final prediction. In this work, the RF contained 50 decision trees with a minimum leaf size of 3 for each tree.

Adaptive boosting (AdaBoost) is an ensemble method often used to obtain satisfactory results compared with other methods [38]. It aims at converting a set of weak classifiers into a strong one. In this work, AdaBoost included 50 shallow decision trees.

GBDT is a machine learning technique comprised of a collection of decision trees to form a stronger prediction model. GBDT builds the model in a stage-wise fashion and trains it iteratively. It implements generalization by allowing optimization of an arbitrary differentiable loss function, which makes them efficient, accurate, and interpretable. Light gradient boosting (LGBM) is a tree-based learning method that implements GBDT [51]. It is suitable for the large size of data and a large number of features. It trains fast, utilizes low memory, and its accuracy is better. Unlike other methods that follow level-wise training pattern, LGBM and XGB follow a leaf-wise training approach [52].

XGB is an advanced GBDT method designed for speed, flexibility, and accuracy [53]. This tree ensemble model is trained in an adaptive manner. It has been used to build a prediction model for protein-protein interface residues [54]. In this work, the features for XGB were selected by its internal algorithm. Features for the classification models were selected by a backward elimination manner. The labels used by the classification methods for prediction were determined by stacked AE. The classification models were then used to test accuracy and robustness in test sets. Since our dataset is a pair-wise sample, the feature combinations with the highest predictive accuracy were selected and fed into an ensemble of the two models to evaluate the performance. Five-fold cross validation technique was used to evaluate the predictive ability of the proposed method. A hybrid method was employed, which combined RF and LGBM classifiers. These classifiers cannot directly work using raw sequence data. Therefore, an unsupervised DL model is implemented for extraction of features. We employ majority voting, a widely used method for the fusion of multiple classifiers.

#### 2.4.5. Experimental Setup

In this study, we used *Arabidopsis thaliana* and *Zea mays* sequence datasets. The source codes for the experiments were written in python 2.7 programming language. For each dataset, we train our models using Keras library (https://github.com/fchollet/keras) with Tensorflow [55] as the backend. We separate the data in the ratio 4:1. Each time, four folds are used as the training set and one is withheld as the test set. We feed the training set into our models and use 20% as a test set. During the training process, test data are used to monitor convergence at the end of each epoch. Adding the number of neurons per layer indicates increasing the number of feature maps. In this experiment, we fix the depth of network to 3. We stack a combination of batch normalization, ReLU layer, and dropout layer to form the encoder. We use three different values for the learning rate (0.5, 1, 2), as shown in Table 2, and the dropping probability 0.6. The momentum update method [56] was employed with a momentum coefficient of 0.9. During the training, we set the maximum number of iterations to 50 for all the training examples. For each iteration, training examples are randomly shuffled to speed up the training process. We set a mini-batch training with a batch size of 64 (*m* = 64).

The model is fine-tuned by back-propagation using categorical cross-entropy loss function, which is optimized by SGD with momentum. Other hyper-parameter values that were optimized during training were the learning decay rate (1e-6) and a mean squared error (MSE). Mixed-norm constraints (*l*_1_ = 0.01 and *l*_2_ = 0.01) are used to minimize reconstruction error. We exploit non-linear activation function on hidden neurons to combat nonlinearity in lncRNA-protein interactions. Encoder function uses ReLU activation function while the decoder function uses sigmoid activation function.

**Figure 3:**
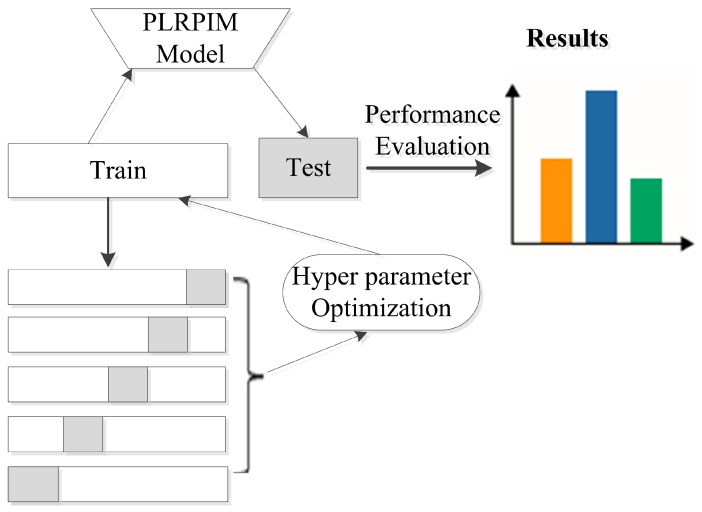
Experimental setup for testing the proposed method. The datasets are split into five folds and hyper parameters are tuned in the training set. The model is learned and applied on the test set based on the hyper parameters. Performance metrics are calculated for all folds.

Additionally, we employ batch normalization and early stopping regularization techniques to avoid overfitting. More details on parameter settings for our model are shown in Table 2. We observe that initially, the performance of PLRPIM increases with the depth of the network. However, the performance drops when the depth of the network is greater than three. This is due to overfitting since increasing the number of hidden layers decreases the training data. Figure 3 summarizes the steps followed during the testing of the proposed method.

#### 2.4.6. PLRPIM Method

PLRPI predicts based on the harmonious blend of information from concatenation fusion of sparse representations of lncRNA and protein sequences. The pseudocode of the proposed method is presented as follows (Algorithm 1).

  Algorithm 1: Pseudo code of **PLRPIM**  **Input:**
*Y*—adjacency matrix of interacting lncRNA *i* and protein *j*; (*Y*(*l_i_, p_j_*)), *S*_lncRNA_—set of lncRNA sequence of interest, *S*_protein_—set of protein sequence of interest, number of stacked AEs = *T,* number of iterations (epoch) = *R*
  **Output:** lncRNA-protein interaction matrix *M_ij_* 1.B = {1, 0}—Binary domain; 2.Interact: *S*_lncRNA_ × *S*_protein_→ B; 3.Interact: (*S(l_i_*), *S(p_i_*))- check whether lncRNA *l_i_* and protein *p_i_* interact; if true, return 1; otherwise, return 0; 4.Concatenate: *S*_lncRNA_ × *S*_protein_→*Y;* 5.Initialize training examples labels (*Y*(*l_i_*, *p_j_*)) = 0; 6.**For***t* = 1 to *T*
**do;** 7.**For***r* = 1 to *R*
**do;** 8.Minimize objective function using formula (10); 9.Compute number of features from similarity matrices ${\left\{X_{i}\right\}}_{i=1}^{m}$ and ${\left\{W_{i}\right\}}_{i=1}^{n}$; 10.Compute hidden vector ${\left\{Z_{i}\right\}}_{n=1}^{N}$ with learned AE; 11.Update ${\left\{Z_{i}\right\}}_{n=1}^{N}$; 12.
**End for;**
 13.Compute feature vectors {*F_l_*_1_, *F_l_*_2_,…, *F_lm_*} and {*F_p_*_1_, *F_p_*_2_,…, *F_pn_*}; 14.Predict class labels of the test dataset based on ensemble voting process using formula (11); 15.Update training examples labels (*Y*(*l_i_*, *p_j_*)) = 1; 16.
**End for;**
 17.
**Return:**
*M_ij._*


### 2.5. Evaluation of PLRPIM

To empirically assess the prediction capability of the proposed model, we adopt 5-fold cross-validation as well as some commonly used measures. Cross-validation iteratively partitions the dataset into training and test sets to reduce sampling bias. The data were randomly split into five sets of equal size. One fold is withheld as the test set and the remaining four are used as the training set. We use six standard metrics including area under ROC curve (AUC), precision (PRE), sensitivity (SEN), specificity (SPEC), accuracy (ACC), and Mathews correlation coefficient (MCC) to evaluate the average performance. The probability of assigning a high rank to a randomly chosen positive instance over a negative one is plotted on the AUC curve. The greater the value of AUC, the better the predictive power of the model. The outcomes from a classifier can be represented as a confusion matrix. The confusion matrix contains four categories of outcomes, true positive (TP), true negative (TN), false positive (FP), and false negative (FN). TP are actual lncRNA-protein pairs that are predicted correctly, TN are the pairs correctly extracted from the negative samples, FP are negative entities falsely predicted, whereas FN are the cases where actual lncRNA-protein pairs are predicted as non-interacting.

(12) $$PRE=\frac{TP}{TP+FP}$$

(13) $$SEN=\frac{TP}{TP+FN}$$

(14) $$SPE=\frac{TN}{TN+FP}$$

(15) $$ACC=\frac{TP+TN}{TP+TN+FP+FN}$$

(16) $$MCC=\frac{TP\times TN-FP\times FN}{\sqrt{\left(TP\;+\;FP)(TP+FN)(TN+FP)(TN+FN\right)}}$$

## 3. Results

In this section, we present 5-fold cross-validation results of PLRPIM on two datasets. Table 3 shows the results obtained from each of the five folds, the average value, and the standard deviation. 

The proposed method has the highest AUC followed by specificity, also referred to as true negative rate. This indicates that our method identifies the percentage of actual non-interactive pairs more accurately for both datasets. Additionally, the standard deviation values showing the variation in the values obtained by each fold are close to zero, indicating low-variance. This shows the uniformity in the results, thus evidencing that our method has reliable performance.

### 3.1. Comparison between Classification Models

We applied six classification methods individually including SVM, AdaBoost, DT, XGB, LGBM, DT, and RF. Then, we presented the average prediction results obtained from 5-fold cross-validation technique. SVM does not perform well compared with the other methods in our experiments. Therefore, we do not include it in the results presented in Table 4. The strength of our pipeline is based on a heterogeneous ensemble strategy, which effectively reduces the false positive rate and achieves better performance. Integration of baseline models trained by different high-level feature combinations boosted the performance.

We compare our multi-model ensemble method with other classification methods, as presented in Table 4. Results obtained indicate that the proposed method increases prediction accuracy for the datasets compared with single classifiers. From the results presented in Table 4, our method performed better in terms of accuracy, precision, specificity, MCC, and AUC for the *Arabidopsis thaliana* dataset. LGBM had the highest sensitivity. For the *Zea mays* dataset, PLRPIM had the highest accuracy, precision, sensitivity, specificity, MCC, and AUC.

**Figure 4:**
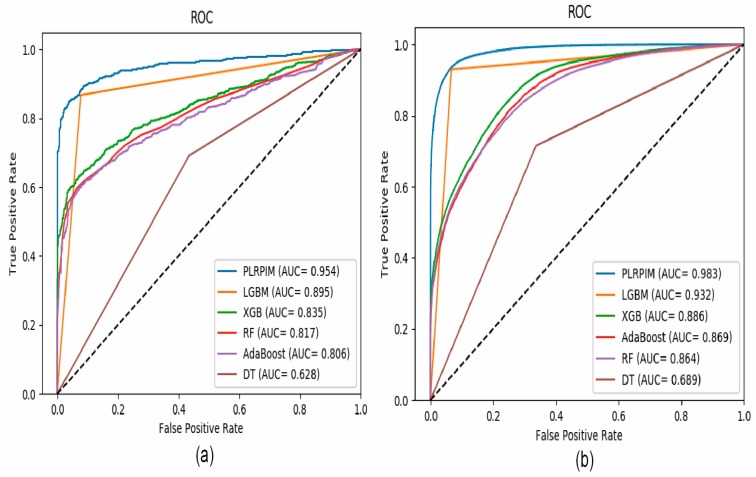
ROC curves for PLRPIM, light gradient boosting machine (LGBM), extreme gradient boost (XGB), random forest (RF), adaptive boosting (AdaBoost), and decision tree (DT) on (**a**) *Arabidopsis thaliana* dataset and (**b**) *Zea mays* dataset.

Figure 4a shows that our method had better performance in terms of AUC on the *Arabidopsis thaliana* dataset. In comparison to the other methods, our method had approximately 15% better performance. The contribution of the two models integrated in PLRPIM significantly boosted the performance. Figure 4b shows that our method had better performance in terms of AUC on the *Zea mays* dataset. In comparison to the other methods, our method had approximately 13% better performance. The performance of all the methods increased in terms of AUC in the *Zea mays* dataset due to the substantial increase in the size of the dataset.

### 3.2. Comparison of PLRPIM with Other Existing Tools

**Figure 5:**
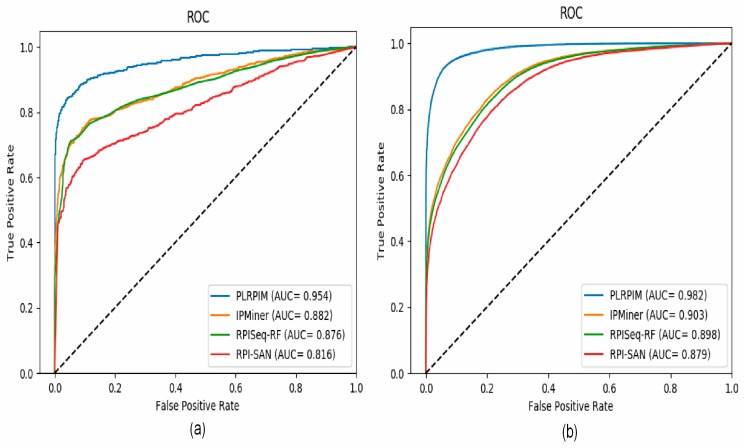
ROC curves for PLRPIM, IPMiner, RPISeq-RF, and RPI-SAN on (**a**) *Arabidopsis thaliana* and (**b**) *Zea mays* datasets.

To test the reliability of the proposed method, we compare PLRPIM with three other sequence-based computational models, RPISeq-RF, RPI-SAN, and IPMiner. The performance of each tool in terms of accuracy, precision, sensitivity, specificity, and AUC was compared. In terms of accuracy, PLRPIM performed better with 89.98% and 93.44% accuracy for the two plants. The AUC values of PLRPIM, IPMiner, RPISeq-RF, and RPI-SAN for *Arabidopsis thaliana* are 0.9546, 0.8823, 0.8761, and 0.8164, respectively, as shown in Figure 5a. For the *Zea mays* dataset, the AUC values are 0.9823, 0.9034, 0.8980, and 0.8792, respectively, as shown in Figure 5b. Table 5 shows the performance of the four methods.

By tapping on the performance benefits of sparsity constraints, our model learns the most informative sequence features. In Table 5, our method performs better that the other methods in terms of accuracy, precision, sensitivity, specificity, MCC, and AUC for both *Arabidopsis thaliana* and *Zea mays* datasets.

Figure 5a shows that our method has better performance in terms of AUC on the *Arabidopsis thaliana* dataset. In comparison to the other methods, our method had approximately 9% better performance in terms of AUC. Figure 5b shows that our method has better performance in terms of AUC on the *Zea mays* dataset. In comparison to the other methods, our method has approximately 8% increase in AUC. Increasing the amount of data positively affects the performance of the model. The better performance is attributed to data size and employing LGBM. LGBM model boosts prediction power since it is suitable for large size of data. All methods are positively influenced by the increase in data size. Their performance in terms of AUC increase.

### 3.3. Functional Analysis of lncRNAs

We explore *Arabidopsis thaliana* and *Zea mays* lncRNA-protein networks for annotation of lncRNAs. For the *Arabidopsis thaliana,* TCONS_00011717, TCONS_00008833, and TCONS_00012080 interact with proteins IPR012677 and P41377. IPR012677 functions include oxidoreductase activity, nucleotide binding, and nucleotide acid binding. P41377 functions include ATP binding, mRNA binding, and helicase activity. In our *Zea mays* dataset, some genes lack annotations and are referred to as “orphan genes”. According to Arendsee et al., orphan genes are defined as genes whose coding sequences are specific to species [57]. Many orphan genes functions are unknown due to lack of homologs and their uniqueness to specific species. Therefore, identifying their protein interaction partners is an alternative way of annotating them. Some orphan genes without functions include GRMZM5G870099, GRMZM2G562000, GRMZM2G046326, GRMZM5G849473, GRMZM2G107204, and GRMZM2G375531.

As an illustration, we provide Table 6 to show the significance of the interaction between lncRNAs and proteins in plant development and regulation of gene expression. For example, the above-mentioned orphan genes and GRMZM2G374777 (orphan gene), GRMZM2G097084 (orphan gene), GRMZM2G078523, GRMZM2G543070, and GRMZM2G147020 interact with protein B6SIF0. This protein has abiotic stress-related functions such as response to cold, response to water deprivation, and response to osmotic stress. We obtain GO annotation terms from EnsemblPlants database (https://plants.ensembl.org) and maize genetics and genomics database (https://maizegdb.org). We observe that the lncRNAs interacting with the same protein share common functions such as two-leaf expansion stage as shown in Table 6.

## 4. Conclusions

In this paper, we presented PLRPIM, a hybrid method for the prediction of plant lncRNA and protein interactions. The proposed method consists of CSAE for feature selection and reduction of feature space dimension, a feed forward, and ensemble classifiers for prediction. The prediction performances demonstrate its efficiency in comparison to other methods. From our results, the hybrid of heterogeneous ensemble classifiers and unsupervised learning implemented by the proposed method performs well without dataset size limitation. By fully exploiting multiple classifiers, the proposed method is shown to have a high success rate for lncRNA-protein interaction prediction based on genome sequence. Additionally, the proposed method is versatile and can be used to predict lncRNA-protein interaction for other plants. However, the proposed method has several potential limitations that need to be addressed in the future. First, the degree of research for plant lncRNA-related proteins for different plant species is limited; thus, known lncRNA-binding protein partners are sparse. Information bias can mislead the measurement of interaction probability between lncRNAs and proteins in plants. More data sources with experimentally validated data can potentially improve the performance further.

## Figures and Tables

**Table 1:** Details of lncRNA–protein interaction datasets for two species.

Species	Dataset	Positive Samples	Negative Samples	Total
*Arabidopsis thaliana*	Training set	758	758	1516
	Test set	190	190	380
*Zea mays*	Training set	17,706	17,706	35,412
	Test set	4427	4427	8854

**Table 2:** Hyper parameter settings.

Name	Settings
Learning rate	0.5, 1, 2
Parameter optimization	SGD, momentum, Adam
Batch size	256, 128, 64
Activation	ReLU
Loss	MSE, Cross Entropy
Dropout rate	0.6

**Table 3:** 5-fold results of the proposed method.

Dataset	Test Set	ACC	PRE	SEN	SPE	MCC	AUC
*Arabidopsis thaliana*	1	0.9000	0.9250	0.8506	0.9417	0.7995	0.9527
	2	0.8865	0.9368	0.8359	0.9402	0.7784	0.9488
	3	0.8971	0.9344	0.8636	0.9337	0.7970	0.9590
	4	0.9050	0.9353	0.8641	0.9436	0.8117	0.9490
	5	0.9103	0.9223	0.9036	0.9176	0.8206	0.9615
	Average	0.8998 ± 0.008	0.9308 ± 0.006	0.8636 ± 0.02	0.9354 ± 0.009	0.8015 ± 0.01	0.9542 ± 0.005
*Zea mays*	1	0.9372	0.9394	0.9338	0.9405	0.8744	0.9849
	2	0.9340	0.9380	0.9313	0.9369	0.8681	0.9846
	3	0.9312	0.9318	0.9303	0.9321	0.8624	0.9817
	4	0.9310	0.9350	0.9259	0.9361	0.8620	0.9812
	5	0.9387	0.9367	0.9408	0.9366	0.8773	0.9833
	Average	0.9344 ± 0.003	0.9362 ± 0.003	0.9324 ± 0.005	0.9364 ± 0.003	0.8689 ± 0.006	0.9831 ± 0.001

**Table 4:** Predictive performance of classifiers and the proposed method.

Dataset	Method	ACC	PRE	SEN	SPE	MCC	AUC
*Arabidopsis thaliana*	PLRPIM	0.8998	0.9308	0.8636	0.9354	0.8015	0.9542
	LGBM	0.8950	0.9182	0.8668	0.9230	0.7912	0.8949
	XGB	0.7452	0.7615	0.7661	0.7187	0.4993	0.8352
	RF	0.7088	0.6851	0.8164	0.5951	0.4294	0.8171
	AdaBoost	0.6962	0.6829	0.8234	0.5622	0.4214	0.8061
	DT	0.6233	0.6331	0.6060	0.6359	0.2478	0.6284
*Zea mays*	PLRPIM	0.9344	0.9362	0.9324	0.9364	0.8688	0.9831
	LGBM	0.9317	0.9331	0.9300	0.9333	0.8634	0.9317
	XGB	0.7936	0.7689	0.8426	0.7446	0.5909	0.8862
	AdaBoost	0.7849	0.7676	0.8182	0.7516	0.5725	0.8693
	RF	0.7536	0.7407	0.7972	0.7103	0.5111	0.8641
	DT	0.6523	0.6500	0.6676	0.6368	0.3049	0.6894

**Table 5:** Predictive performance of other methods and the proposed method.

Dataset	Method	ACC	PRE	SEN	SPE	MCC	AUC
*Arabidopsis thaliana*	PLRPIM	0.8998	0.9308	0.8636	0.9354	0.8015	0.9546
	IPMiner	0.8275	0.8930	0.7448	0.9107	0.6646	0.8823
	RPISeq-RF	0.8059	0.8144	0.7922	0.8200	0.6124	0.8761
	RPI-SAN	0.7579	0.7955	0.6966	0.8199	0.5210	0.8164
*Zea mays*	PLRPIM	0.9344	0.9362	0.9324	0.9364	0.8688	0.9823
	IPMiner	0.8127	0.8142	0.8106	0.8148	0.6258	0.9034
	RPISeq-RF	0.8069	0.7993	0.8192	0.7945	0.6142	0.8980
	RPI-SAN	0.7890	0.7909	0.7869	0.7911	0.5784	0.8792

**Table 6:** Some selected *Zea mays* and *Arabidopsis thaliana* long non-coding RNAs (lncRNAs) and their biological functions.

Species	lncRNAs	Biological Functions
*Arabidopsis thaliana*	TCONS_00011717	GO:0006913—Nucleocytoplasmic transport
	TCONS_00008833	GO:0006083—Acetate metabolic process
	TCONS_00012080	GO:0009867—Regulation of jasmonic acid mediated signaling pathway
*Zea mays*	GRMZM2G374777	PO:0001052—2 leaf expansion stage PO:0006339—juvenile vascular leaf PO:0009089—endosperm
	GRMZM2G097084	GO:0005524—ATP binding GO:0006183—GTP biosynthetic process GO:0006952—defense response
	GRMZM2G078523	PO:0001052—2 leaf expansion stage PO:0009084—pericarp PO:0009089—endosperm
	GRMZM2G543070	PO:0001052—2 leaf expansion stage PO:0001095—true leaf formation stage PO:0007016—4 flowering stage
	GRMZM2G147020	PO:0001052—2 leaf expansion stage PO:0001095—true leaf formation stage PO:0007016—4 flowering stage PO:0001009—D pollen mother cell meiosis stage
